# Effects of nitric oxide-releasing nanoparticles on neotropical tree seedlings submitted to acclimation under full sun in the nursery

**DOI:** 10.1038/s41598-019-54030-3

**Published:** 2019-11-22

**Authors:** Patrícia Juliana Lopes-Oliveira, Diego Genuário Gomes, Milena Trevisan Pelegrino, Edmilson Bianchini, José Antonio Pimenta, Renata Stolf-Moreira, Amedea Barozzi Seabra, Halley Caixeta Oliveira

**Affiliations:** 1 Department of Animal and Plant Biology, Universidade Estadual de Londrina (UEL), Rodovia Celso Garcia Cid, km 380, CEP 86057-970 Londrina, PR Brazil; 2 Center for Natural and Human Sciences, Universidade Federal do ABC (UFABC), Av. dos Estados 5001, CEP 09210-580 Santo André, SP Brazil

**Keywords:** Plant physiology, Nanoparticles

## Abstract

Polymeric nanoparticles have emerged as carrier systems for molecules that release nitric oxide (NO), a free radical involved in plant stress responses. However, to date, nanoencapsulated NO donors have not been applied to plants under realistic field conditions. Here, we verified the effects of free and nanoencapsulated NO donor, S-nitroso-mercaptosuccinic acid (S-nitroso-MSA), on growth, physiological and biochemical parameters of neotropical tree seedlings kept under full sunlight in the nursery for acclimation. S-nitroso-MSA incorporation into chitosan nanoparticles partially protected the NO donor from thermal and photochemical degradation. The application of nanoencapsulated S-nitroso-MSA in the substrate favoured the growth of seedlings of *Heliocarpus popayanensis*, a shade-intolerant tree. In contrast, free S-nitroso-MSA or nanoparticles containing non-nitrosated mercaptosuccinic acid reduced photosynthesis and seedling growth. Seedlings of *Cariniana estrellensis*, a shade-tolerant tree, did not have their photosynthesis and growth affected by any formulations, despite the increase of foliar S-nitrosothiol levels mainly induced by S-nitroso-MSA-loaded nanoparticles. These results suggest that depending on the tree species, nanoencapsulated NO donors can be used to improve seedling acclimation in the nursery.

## Introduction

Seedlings of neotropical trees are subjected to numerous abiotic stresses when transplanted to deforested areas, which may affect their survival in the field^1–3^. Hence, neotropical seedlings need to pass through the acclimation or hardening process in the nursery, which is usually achieved by the growth of the seedlings under full sun^4–7^. During this process, plants are exposed to multiple abiotic stress factors, such as high irradiance, ultraviolet (UV) radiation and high temperature. This approach thus improves the plant stress responses, resulting in more tolerant seedlings and decreased replanting needs^8–11^. In this scenario, the development of management techniques that favour the acclimation process in the nursery is of great relevance for the production of high-quality seedlings.

Nitric oxide (NO) has attracted considerable interest from the scientific community due to its importance as a signalling molecule in plant responses to stresses^12–14^. Studies have shown that NO increases plant tolerance to abiotic stresses, including drought, salinity, heavy metals and extreme temperatures^12,14–16^. NO is also involved in signalling pathways that regulate antioxidant mechanisms, protecting plants against oxidative stress induced by high light intensities and UV radiation^16–19^.

However, direct treatment of plants with NO is a laborious method due to its gaseous nature, demanding the use of specific equipment^20,21^. Moreover, NO has a short half-life (<6 s), making the constant NO supply to tissues necessary^22^. Low-molecular-weight NO donors, such as sodium nitroprusside (SNP) and S-nitrosothiols (RSNO), have been widely used in studies with different living organisms^19,23^. For instance, the application of SNP in nutrient solution improved the antioxidant response and prevented the aluminum-induced cell death in *Arachis hypogaea* roots^24^. In another study, the treatment of *Saccharum* spp. leaves with S-nitrosoglutathione (GSNO) alleviated the negative effects of drought on photosynthesis^25^. In addition to stress protection, different NO donors can be applied for other agricultural purposes, such as the control of seed germination and fruit ripening^26^. Nevertheless, NO donors are relatively unstable and susceptible to thermal and photochemical decomposition, which may cause a rapid NO release, resulting in reduced signalling effects^22^.

The encapsulation of NO donors using nanomaterials has emerged as a strategy to protect these molecules from decomposition, allowing controlled NO release and extending its period of action^22,27^. Several polymeric nanoparticles (NPs) have been successfully developed as carrier systems for NO donors in biomedicine^20,27^. Among these particles, NPs that are composed of chitosan, a biodegradable and mucoadhesive polymer, have low toxicity and facilitate cell absorption^28^.

Despite this method’s numerous potential applications on plant growth, seed germination and stress response, the use of NO-releasing NPs in plant science has been only recently developed^29^. Only in 2016, Oliveira *et al*.^30^ reported that the treatment with chitosan NPs loaded with S-nitroso-mercaptosuccinic acid (S-nitroso-MSA, a NO donor that belongs to the class of S-nitrosothiols) protected maize plants against the effects of salt stress. In addition, encapsulated S-nitroso-MSA was more efficient than the free NO donor in inducing tolerance to salinity. The improvement in NO bioactivity was associated with sustained NO release by polymeric NPs^30^. This pioneering work opened a range of possibilities for the application of NO-releasing NPs in plants.

More recent studies have demonstrated the effectiveness of GSNO-loaded chitosan NPs in increasing the tolerance of sugarcane plants to drought stress and in preserving the quality of cherry fruits during cold storage^31,32^. Therefore, the treatment with NO-releasing NPs has emerged as a strategy to obtain plants that are more tolerant to abiotic stresses. In particular, it can be hypothesized that NO-releasing NPs may help neotropical tree seedlings to acclimate under full sun in the nursery.

The objective of this study was to verify the effects of free and nanoencapsulated S-nitroso-MSA on growth, physiological and biochemical parameters of *Heliocarpus popayanensis* Kunth and *Cariniana estrellensis* (Raddi) Kuntze seedlings kept under full sunlight in the nursery for acclimation. To the best of our knowledge, this report is the first to describe the positive effects of NO-releasing NPs on plants growing under realistic nursery conditions.

## Results

### NP characterization and kinetics of NO release

The encapsulation efficiency of MSA into NPs was found to be 91.07 ± 0.01%. This high value indicates the successful formation of MSA NPs, which is attributed to the strong electrostatic interactions of MSA with chitosan chains^33^. Dynamic light scattering (DLS) measurements revealed that the average hydrodynamic size, polydispersity index (PDI) and zeta potential of MSA NPs were 37.92 ± 2.02 nm, 0.345 ± 0.009, and +17.5 ± 0.9 mV, respectively. These results indicate that the particles have a nanoscale size with low to moderate dispersion and homogeneous size distribution in aqueous medium. The positive zeta potential is attributed to the presence of protonated amino groups in the chitosan chain, indicating the formation of a stable colloidal suspension. These results are in accordance with our previous work, in spite of the difference of MSA concentration in the NPs^30^.

**Figure 1:**
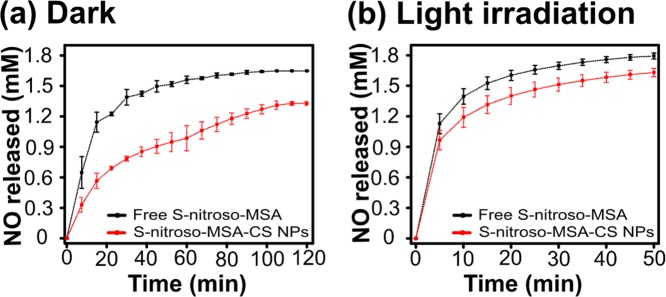
Kinetic curves of NO release from free S-nitroso-MSA (black curves) and S-nitroso-MSA loaded in chitosan nanoparticles (red curves), at 38.5 °C, (**a**) in the dark and (**b**) under white-light irradiation. In both cases, the initial concentration of S-nitroso-MSA was 2 mM. Traces on the points correspond to the standard errors (n = 2).

Figure 1 shows the kinetic curves for NO release of free and encapsulated S-nitroso-MSA at high temperature in the dark (a) and under white light irradiation (b). It can be observed that all curves have two stages: (i) an initial burst in the NO release (in the first 50 min and 15 min for the curves in the dark and under white light irradiation, respectively) (ii) followed by a second stage with the establishment of a steady state. In the dark, the initial rates of NO release from free and encapsulated S-nitroso-MSA were 0.035 ± 0.008 and 0.022 ± 0.003 mmol L^−1^ min^−1^, respectively. In contrast, under white light irradiation, the initial rates of NO release from free and encapsulated S-nitroso-MSA were found to be 0.139 ± 0.049 and 0.118 ± 0.042 mmol L^−1^ min^−1^, respectively. Thus, the encapsulation of the NO donor into chitosan NPs decreased the initial rates of NO release by 50 and 20% in the dark and under white light irradiation, respectively.

The average concentrations of NO released at steady-state in the dark were 1.64 ± 0.03 and 1.31 ± 0.03 mM for free and encapsulated S-nitroso MSA, respectively. Similarly, the average concentrations of NO released at steady state under white light irradiation were 1.76 ± 0.03 and 1.59 ± 0.03 mM for free and encapsulated S-nitroso MSA, respectively. The encapsulation of the NO donor decreased the steady-state concentration by 25% in the dark and 10% under white-light irradiation in comparison with free S-nitroso-MSA under the same conditions. Overall, the results indicate that chitosan NPs have a protective effect on the thermal and photochemical stability of S-nitroso-MSA.

### Growth measurements

S-nitroso-MSA NPs treatment improved the growth of *H. popayanensis* seedlings compared to control seedlings (Table 1). The application of S-nitroso-MSA NPs promoted the increase of the following growth parameters: leaf area (increase of 31.4%), stem length and diameter (23.5% and 8.6% increases, respectively), and leaf and stem dry masses (28.6% and 25.8% increases, respectively), compared to the control group.

**Table 1:** Mean ± standard error (n = 12) of growth parameters of *Heliocarpus popayanensis* (*Hp*) and *Cariniana estrellensis* (*Ce*) seedlings grown under full sun and submitted to the following treatments: Control = no treatment; S-nitroso-MSA NPs = chitosan nanoparticles containing the NO donor; S-nitroso-MSA = non-encapsulated NO donor; and MSA NPs = chitosan nanoparticles containing MSA. SLA = specific leaf area; DQI = Dickson quality index; H/D = stem height/stem diameter ratio; S/R = shoot-to-root dry mass ratio. Means followed by the same letter in the row did not differ by ANOVA followed by the Tukey test (p < 0.05).

Parameters	Species	Treatment			
		Control	S-nitroso-MSA NPs	S-nitroso-MSA	MSA NPs
Leaf area (cm^2^)	*Hp*	43.54 ± 1.78^**b**^	57.20 ± 3.54^**a**^	38.01 ± 2.38^**b**^	34.93 ± 1.71^**b**^
	*Ce*	145.12 ± 9.05^**a**^	130.50 ± 8.34^**a**^	121.35 ± 7.05^**a**^	129.41 ± 8.69^**a**^
Stem height (cm)	*Hp*	13.36 ± 0.56^**b**^	16.5 ± 0.58^**a**^	12.4 ± 0.53^**b**^	11.46 ± 0.34^**b**^
	*Ce*	14.07 ± 0.69^**a**^	13.42 ± 0.36^**a**^	13.84 ± 0.55^**a**^	14.59 ± 0.62^**a**^
Stem diameter (mm)	*Hp*	4.08 ± 0.09^**b**^	4.43 ± 0.10^**a**^	4.01 ± 0.09^**b**^	3.52 ± 0.06^**c**^
	*Ce*	4.14 ± 0.11^**a**^	3.80 ± 0.07^**a**^	3.80 ± 0.09^**a**^	4.14 ± 0.17^**a**^
Leaf dry mass (g)	*Hp*	0.21 ± 0.005^**b**^	0.27 ± 0.01^**a**^	0.17 ± 0.01^**c**^	0.15 ± 0.007^**c**^
	*Ce*	0.63 ± 0.05^**a**^	0.53 ± 0.03^**a**^	0.53 ± 0.03^**a**^	0.55 ± 0.04^**a**^
Stem dry masss (g)	*Hp*	0.31 ± 0.02^**b**^	0.39 ± 0.02^**a**^	0.27 ± 0.02^**bc**^	0.21 ± 0.01^**c**^
	*Ce*	0.43 ± 0.03^**ab**^	0.33 ± 0.02^**b**^	0.37 ± 0.02^**ab**^	0.45 ± 0.04^**a**^
Root dry mass (g)	*Hp*	0.25 ± 0.01^**ab**^	0.29 ± 0.01^**a**^	0.24 ± 0.01^**bc**^	0.20 ± 0.01^**c**^
	*Ce*	0.40 ± 0.04^**a**^	0.28 ± 0.03^**a**^	0.36 ± 0.04^**a**^	0.41 ± 0.06^**a**^
S/R ratio (g g^−1^)	*Hp*	2.08 ± 0.16^**ab**^	2.23 ± 0.14^**a**^	1.84 ± 0.12^**b**^	1.89 ± 0.04^**b**^
	*Ce*	2.62 ± 0.14^**a**^	3.13 ± 0.11^**a**^	2.64 ± 0.11^**a**^	3.02 ± 0.13^**a**^
H/D ratio (cm mm^−1^)	*Hp*	1.93 ± 0.16^**a**^	2.05 ± 0.14^**a**^	1.89 ± 0.12^**a**^	1.92 ± 0.04^**a**^
	*Ce*	3.29 ± 0.14^**a**^	3.59 ± 0.11^**a**^	3.64 ± 0.11^**a**^	3.43 ± 0.13^**a**^
DQI	*Hp*	0.15 ± 0.008^**ab**^	0.17 ± 0.01^**a**^	0.13 ± 0.005^**bc**^	0.11 ± 0.004^**c**^
	*Ce*	0.24 ± 0.02^**a**^	0.18 ± 0.02^**a**^	0.21 ± 0.02^**a**^	0.25 ± 0.03^**a**^
SLA (cm^2^ g^−1^)	*Hp*	212.77 ± 3.55^**b**^	224 ± 6.5^**ab**^	230.13 ± 5.60^**ab**^	237.51 ± 3.87^**a**^
	*Ce*	229.50 ± 6.24^**a**^	240.20 ± 3.77^**a**^	228 ± 4.85^**a**^	234.24 ± 6.59^**a**^

In contrast, free S-nitroso-MSA and MSA NPs treatments did not have positive effects on the growth of *H. popayanensis* seedlings. Some parameters were even reduced in relation to the control, mainly by the MSA NPs treatment. For example, stem diameter and Dickson quality index (DQI) decreased by 13.7 and 26.7%, respectively, in MSA NPs-treated plants relative to the control. There were also reductions induced by MSA NPs treatment on leaf, stem and root dry masses (decreases of 28.6%, 32.3% and 20%, respectively, compared with the control). For S-nitroso-MSA, only a decrease of 19.1% in leaf dry mass was observed.

For *C. estrellensis* seedlings, the treatments with S-nitroso-MSA NPs, S-nitroso-MSA and MSA NPs did not induce any significant effects on the evaluated growth parameters in relation to the control (Table 1).

### Physiological and biochemical measurements

**Figure 2:**
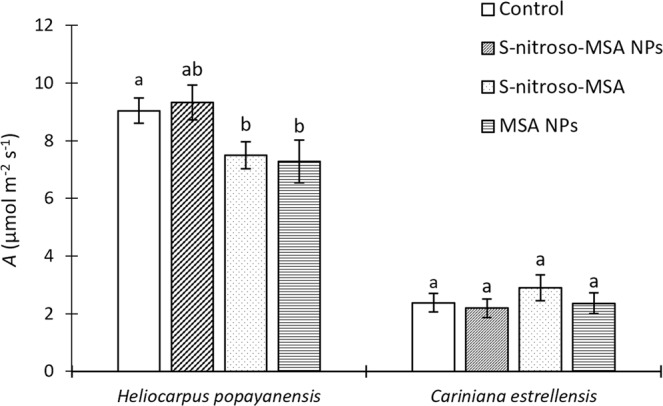
Net photosynthesis (*A*) of *Heliocarpus popayanensis* and *Cariniana estrellensis* seedlings grown under full sun and submitted to the following treatments: Control = no treatment (white bar); S-nitroso-MSA NPs = chitosan nanoparticles containing the NO donor (bar with diagonal lines); S-nitroso-MSA = non-encapsulated NO donor (dotted bar); and MSA NPs = chitosan nanoparticles containing MSA (dashed bar). Treatments with equal letters do not differ by ANOVA followed by the Tukey test (p < 0.05). Traces on the bars correspond to the standard error (n = 10).

For *H. popayanensis*, no increase was observed in net photosynthesis (*A*) for plants treated with S-nitroso-MSA NPs in comparison to the control group (Fig. 2). For the seedlings treated with free S-nitroso-MSA and MSA NPs, there was a decrease of *A* in comparison with the control group. The treatments did not have any significant effects on *A* of *C. estrellensis* seedlings.

S-nitroso-MSA NPs and MSA NPs treatments induced a decrease in the content of phenolic compounds in the leaves of *H. popayanensis* compared to the control, whereas the same parameter was increased by S-nitroso-MSA (Table 2). S-nitroso-MSA-treated seedlings also showed increased leaf flavonoid content in relation to the other treatments. The application of S-nitroso-MSA NPs led to an increase in H_2_O_2_ levels in the leaves of *H. popayanensis* compared to the control. Regarding the antioxidant enzymes, the only observed effect was the decrease of leaf superoxide activity (SOD) activity in MSA NPs-treated seedlings. The other biochemical parameters of *H. popayanensis* leaves did not differ between the treatments and the control.

**Table 2:** Mean ± standard error (n = 5) of biochemical parameters of leaves of *Heliocarpus popayanensis* (*Hp*) and *Cariniana estrellensis* (*Ce*) seedlings grown under full sun and submitted to the following treatments: Control = no treatment; S-nitroso-MSA NPs = chitosan nanoparticles containing the NO donor; S-nitroso-MSA = non-encapsulated NO donor; and MSA NPs = chitosan nanoparticles containing MSA.

Parameters	Species	Treatment			
		Control	S-nitroso-MSA NP	S-nitroso-MSA	MSA NP
Phenolic compounds (mg g^−1^ FW)	*Hp*	14.87 ± 0.94^**b**^	13.27 ± 0.25 ^**c**^	19.83 ± 0.45^**a**^	11.42 ± 0.16^**d**^
	*Ce*	59.93 ± 4.49^**a**^	65.99 ± 2.89^**a**^	62.59 ± 4.41^**a**^	49.41 ± 3.51^**a**^
Flavonoids (mg g^−1^ FW)	*Hp*	23.47 ± 1.47^**b**^	23.17 ± 2.03^**b**^	30.90 ± 1.39^**a**^	19.54 ± 0.75^**b**^
	*Ce*	46.48 ± 2.50^**a**^	52.62 ± 1.95^**a**^	49.22 ± 2.30^**a**^	47.43 ± 4.95^**a**^
APX activity (µmol min^−1^ mg^−1^ protein)	*Hp*	0.68 ± 0.09^**a**^	0.82 ± 0.10^**a**^	0.53 ± 0.07^**a**^	0.79 ± 0.07^**a**^
	*Ce*	1.37 ± 0.28^**ab**^	1.42 ± 0.18^**ab**^	2.34 ± 0.09 ^**a**^	0.88 ± 0.38^**b**^
POD activity (µmol min^−1^ mg^−1^ protein)	*Hp*	6.66 ± 0.42^**a**^	7.76 ± 0.39^**a**^	6.87 ± 0.11^**a**^	6.42 ± 0.37^**a**^
	*Ce*	3.85 ± 0.85^**a**^	3.61 ± 0.78^**a**^	5.73 ± 1.66^**a**^	2.74 ± 0.38^**a**^
SOD activity (U µg^−1^ protein)	*Hp*	2.96 ± 0.08^**a**^	2.74 ± 0.22^**a**^	2.58 ± 0.25^**a**^	1.55 ± 0.09^**b**^
	*Ce*	15.52 ± 3.04^**a**^	12.67 ± 0.47^**a**^	16.71 ± 2.53^**a**^	9.85 ± 1.08^**a**^
H_2_O_2_ (µmol g^−1^ FW)	*Hp*	145.88 ± 8.53^**b**^	167.35 ± 4.70^**a**^	153.23 ± 6.47^**ab**^	150.88 ± 4.12^**b**^
	*Ce*	176.47 ± 20^**b**^	227.94 ± 8.23^**a**^	177.35 ± 40.59^**b**^	192.06 ± 10.59^**ab**^
Chlorophylls (mg g^−1^ FW)	*Hp*	0.62 ± 0.04^**a**^	0.68 ± 0.13^**a**^	0.73 ± 0.07^**a**^	0.73 ± 0.03^**a**^
	*Ce*	2.19 ± 0.13^**a**^	2.70 ± 0.16^**a**^	2.78 ± 0.12^**a**^	3.03 ± 0.16^**a**^
Carotenoids (mg g^−1^ FW)	*Hp*	2.19 ± 0.21^**a**^	2.70 ± 0.30^**a**^	2.78 ± 0.33^**a**^	3.03 ± 0.19^**a**^
	*Ce*	2.71 ± 0.35^**a**^	3.95 ± 0.32^**a**^	10.26 ± 1.48^**a**^	17.94 ± 8.80^**a**^

FW = fresh weight; APX = ascorbate peroxidase; POD = peroxidase; SOD = superoxide dismutase; H_2_O_2_ = hydrogen peroxide. Means followed by equal letters in the line do not differ by ANOVA followed by the Tukey test (p < 0.05).

The treatment of S-nitroso-MSA NPs led to an increase in foliar H_2_O_2_ levels of *C. estrellensis* seedlings compared to the control (Table 2). The other biochemical parameters of *C. estrellensis* leaves did not differ between the treatments and the control.

**Figure 3:**
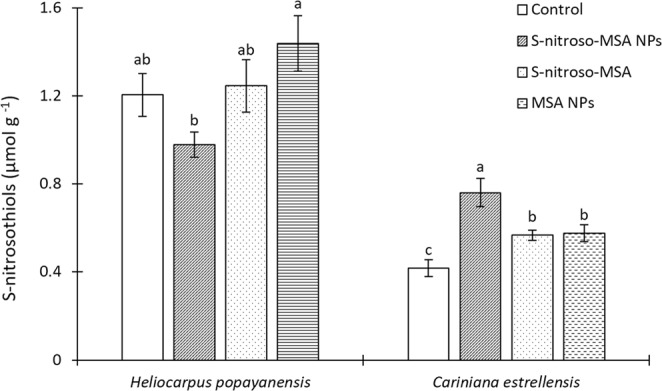
S-nitrosothiol (RSNO) content of leaves of *Heliocarpus popayanensis* and *Cariniana estrellensis* seedlings grown under full sun and submitted to the following treatments: Control = no treatment (white bar); S-nitroso-MSA NPs = chitosan nanoparticles containing the NO donor (bar with diagonal lines); S-nitroso-MSA = non-encapsulated NO donor (dotted bar); and MSA NPs = chitosan nanoparticles containing MSA (dashed bar). Treatments with equal letters do not differ by ANOVA followed by the Tukey test (p < 0.05). Traces on the bars correspond to the standard error (n = 5).

The treatment of *H. popayanensis* plants with the formulations did not alter the leaf RSNO content compared to the control (Fig. 3). In the case of *C. estrellensis*, all treatments induced an increase in leaf RSNO levels in relation to the control. The highest increase in RSNO content was induced by the S-nitroso-MSA NPs treatment.

## Discussion

Many studies have reported the positive effect of NO donors on plants subjected to abiotic stress factors, such as drought, high irradiance, UV radiation and extreme temperatures^12,14–16^. However, few reports have described the application of NO donors for improving the growth of plants growing under realistic field conditions. In this study, we tested the effects of the treatments with free and nanoencapsulated S-nitroso-MSA on the growth, biochemical and physiological parameters of neotropical tree seedlings kept under full sunlight in the acclimation sector of the nursery. We observed an improved growth of seedlings of the shade-intolerant species *H. popayanensis* when treated with S-nitroso-MSA NPs but not when the free NO donor was applied. This result demonstrates the benefit of S-nitroso-MSA nanoencapsulation, enabling the successful application of the NO donor to plants growing in the nursery’s conditions. However, the positive effects of NO on acclimation depend on the tree species, as seedlings of shade-tolerant *C. estrellensis* were considerably less affected by the same NO treatments.

The decomposition of RSNO (such as S-nitroso-MSA), which releases free NO, is strongly favoured by increases in temperature, light intensity, and initial RSNO concentration^34,35^. Thus, free S-nitroso-MSA would probably undergo a fast decomposition when applied to plants in the acclimation sector of the nursery, which could hamper its biological activity. In this study, we showed that the encapsulation of the NO donor into chitosan NPs slightly decreased the rate of S-nitroso-MSA decomposition in the presence of light and at high temperature. However, in the dark and at the same temperature, the encapsulation of the NO donor promoted superior stabilization of S-nitroso-MSA. In other words, the encapsulation effect on the stability of the NO donor was more significant in the dark. Considering that the formulations were applied directly to the substrate, only the fraction of the NO donor that remained near the substrate surface was exposed to significant light intensities. For most of the applied S-nitroso-MSA, temperature was probably the main factor that modulated NO release. An even higher protective effect of nanoencapsulation on S-nitroso-MSA decomposition was detected in previous assays with the same formulation in the dark at room temperature^30^, which is similar to the average temperature during the period of the experiment (see Methods section).

Thus, the slower and more sustained NO release by S-nitroso-MSA NPs compared to the free S-nitroso-MSA is probably related to the fact that the improved growth of *H. popayanensis* seedlings was induced only by the treatment with the encapsulated NO donor. In contrast, the fast NO release by free S-nitroso-MSA may have induced different responses in plant cells, leading to the accumulation of phenolic compounds and flavonoids, inhibition of photosynthesis, and decrease of leaf biomass. It might also be hypothesized that nanoencapsulation may favour the uptake of S-nitroso-MSA by the roots or minimize the leaching of the NO donor in the substrate resulting from the periodic irrigation of the tubes and from frequent atmospheric precipitation.

In contrast to *H. popayanensis*, the administration of S-nitroso-MSA NPs did not affect the growth parameters of seedlings of the shade-tolerant species *C. estrellensis*. Despite this finding, RSNO levels increased in the leaves of S-nitroso-MSA NPs-treated *C. estrellensis* seedlings in comparison with those treated with free S-nitroso-MSA and control seedlings, which indicates the effectiveness of the S-nitroso-MSA NPs treatment in increasing NO bioavailability^36^. Treatment with a lower concentration of encapsulated S-nitroso-MSA (1 mM) did not affect foliar RSNO levels or affect the growth of *C. estrellensis* seedlings (data not shown). Overall, these results demonstrate that the response of *C. estrellensis* seedlings to S-nitroso-MSA NPs differs from that of *H. popayanensis*, indicating that plant response to NO may depend on the species.

The analysis of physiological and biochemical parameters provided some insights into the mechanisms involved in the improved growth of S-nitroso-MSA NPs-treated *H. popayanensis* seedlings. Although S-nitroso-MSA NPs did not affect CO_2_ assimilation per leaf area, this treatment increased the total leaf area of *H. popayanensis*. Consequently, the total CO_2_ assimilation per plant was increased by S-nitroso-MSA NPs, which can be related to the higher biomass accumulation of these seedlings. This characteristic favours rapid plant growth when subjected to high light intensity^37^. The improved growth of *H. popayanensis* seedlings treated with S-nitroso-MSA NPs was not related to an induction of enzymatic antioxidant mechanisms in leaves, as the activity of SOD, POD and APX was not affected by this treatment or by modifications in photosynthetic pigments. However, S-nitroso-MSA NPs increased the H_2_O_2_ content in *H. popayanensis* leaves, a result that was also observed in *C. estrellensis* leaves. Similar to NO, H_2_O_2_ can act as a signalling molecule and induce protective mechanisms and acclimation responses^38^. In addition, these molecules can act together in various signalling pathways to induce cross-resistance to abiotic stress factors^38^.

The comparison of the responses induced by S-nitroso-MSA NPs and MSA NPs treatments suggests that the positive effects of S-nitroso-MSA NPs on *H. popayanensis* seedlings are related to NO/RSNO signalling and not to the other NP constituents. In contrast to S-nitroso-MSA NPs treatment, the administration of non-nitrosated MSA NPs inhibited photosynthesis, SOD activity and most of the analysed growth parameters. These negative effects of MSA NPs on the seedlings are probably related to reduced MSA, as they were not observed when MSA loaded in NPs was nitrosated. Some studies suggest that the increased concentration of compounds with thiol groups can alter the redox balance in living cells, inducing deleterious effects. For instance, high levels of cysteine in *Escherichia coli* promoted oxidative DNA damage by driving the Fenton reaction^39^. Moreover, genetically modified *Nicotiana tabacum* plants with high leaf levels of glutathione also had enhanced oxidative stress, causing a failure of the redox-sensing process in the chloroplast^40^. The accumulation of reduced sulfur compounds such as sulfite and thiosulfate in *Zea mays* plants has also been related to toxic effects^41^. In contrast, chitosan is a biodegradable molecule with low toxicity to plants^42^. Most studies with chitosan report positive effects of this molecule on plant growth and stress responses^43^. However, these results highlight the importance of future studies evaluating the toxicity of these nanoparticles and their subproducts to plants and other organisms, including the soil microbiota^44^.

Despite the beneficial effects of S-nitroso-MSA NPs on the growth of *H. popayanensis* seedlings, this treatment did not result in higher leaf RSNO levels compared to the control. This result likely occurred because we determined leaf RSNO levels at a single time-point, whereas NO/RSNO homeostasis is a dynamic process. The nitrosation of intracellular thiols resulting from S-nitroso-MSA NPs treatment might have induced S-nitrosoglutathione reductase (GSNOR), which is the main enzyme involved in RSNO degradation^45^. In accordance with our results, Kneeshaw *et al*.^46^ reported a time-dependent decay of RSNO content in S-nitroso-cysteine-treated plants. In addition, Ziogas *et al*.^47^ demonstrated that the GSNOR activity in citrus plants is positively modulated by SNP treatment. Oliveira *et al*.^30^ demonstrated lower levels of RSNO in the leaves of maize plants treated with 100 µM of S-nitroso-MSA (free or encapsulated) compared with plants treated with 50 µM of the NO donor. In addition to GSNOR, other proteins, such as thioredoxin, may decrease the excessive RSNO content in plants^47^. It is noteworthy that as formulations were added directly to the substrate, NO/RSNO might have accumulated in the roots, promoting systemic changes that would explain the positive effects obtained on *H. popayanensis* seedlings. Thus, further studies on the NO/RSNO balance and signalling after the administration of free and nanoencapsulated NO donors are still required to unveil the involved mechanisms, including the identification of the molecular targets of NO/RSNO (as S-nitrosated proteins) in different plant organs.

The application of encapsulated NO donors in plant studies is rather recent, and previous studies on this subject have been carried out under plant growth conditions that are more controlled than ours. Oliveira *et al*.^30^ showed that S-nitroso-MSA chitosan NP treatment was more efficient than free S-nitroso-MSA in protecting maize plants against the deleterious effects of salt stress. In this work, the plants were cultivated in sand under relatively controlled greenhouse conditions^30^. Silveira *et al*.^31^ demonstrated the improved efficiency of GSNO-loaded chitosan nanoparticles compared with the free NO donor in protecting hydroponically grown sugarcane plants submitted to drought stress and cultivated in a growth chamber under controlled conditions. In contrast, our study was carried out following the nursery’s routine: the formulations were applied to small-volume tubes containing plant substrate subjected to periodic irrigation and exposed to natural atmospheric conditions. However, the positive effects of S-nitroso-MSA NPs on the growth of *H. popayanensis* seedlings were observed under these unfavourable conditions, suggesting new possibilities for the application of these nanoformulations. It is also noteworthy that S-nitroso-MSA is more cost-effective than other more used donors, such as GSNO and SNPs^23^.

Overall, these results suggest that the application of NO-releasing nanoparticles can be considered as a strategy to improve the acclimation of neotropical trees in the nursery with potential impacts on reforestation programmes. However, the positive effects of S-nitroso-MSA NPs on seedling growth occurred only for *H. popayanensis* seedlings but not for *C. estrellensis*. Further studies are needed to demonstrate whether these results can be extrapolated to other tree species of the same functional groups.

## Methods

### Synthesis of chitosan NPs containing MSA

Chitosan NPs containing 10 mM of mercaptosuccinic acid (MSA, the precursor of the NO donor) were prepared by ionotropic gelation. Chitosan (low-molecular-weight, 75% deacetylation) was purchased from Sigma-Aldrich (St. Louis, MO, USA). Briefly, chitosan (26.7 mg mL^−1^) and MSA (4.0 mg mL^−1^) were dissolved in 1% acetic acid (v/v) under magnetic stirring for 90 min at room temperature (pH ~ 4). A sodium tripolyphosphate (TPP) aqueous solution (2.4 mg mL^−1^) was transferred to tubing attached to an automatic pump controller unit (MPV-500, Marte, São Paulo, Brazil) to be dropped into chitosan/MSA solution (flow rate of 132 µL min^−1^). The added volume of TPP solution followed the volumetric proportion of 3 chitosan/MSA to 1 TPP. The final mixture was further stirred for 60 min at room temperature to allow the formation of chitosan NPs (1000 μg chitosan mL^−1^) containing MSA (10 mM)^33,48^.

### Encapsulation efficiency of MSA into chitosan NPs

The encapsulation efficiency of MSA into chitosan NPs was evaluated by the reaction of free, non-encapsulated MSA with Ellman’s reagent. 5,5′-Dithiobis(2-nitrobenzoic acid) (DTNB) specifically reacts with thiol moieties of MSA forming 2-nitro-5-thiobenzoate anion (TNB^−2^), which has an absorbance peak at 412 nm (ε = 14,150 M^−1^ cm^−1^)^33^. A volume of 500 µL of MSA-containing chitosan NPs was placed into a Microcon centrifugal filter device (MWCO 10,000, Millipore) and centrifuged for 5 min at 112 x*g*. The eluted solution (non-encapsulated MSA) was incubated for 10 min with 2 mL of DTNB solution (0.7 mM) in PBS buffer (pH 7.4) containing EDTA (1 mM), and the absorbance at 412 nm was measured. The measurements were performed in triplicate, and the percentage of encapsulated MSA was calculated using Eq. 1.

1 $$Encapsulation\,efficiency\,(EE \% )=[(\frac{total\,MSA-free\,MSA}{total\,MSA})\times 100]$$

### Dynamic light scattering

The average hydrodynamic diameter (% by intensity), PDI, and zeta potential of MSA-containing chitosan NPs were characterized by DLS (Nano ZS Zetasizer, Malvern Instruments Co, UK). Measurements were performed in triplicate of two independent experiments (n = 6) at 25 °C using a fixed angle of 173° in disposable folded capillary zeta cells with a 10-mm path length in aqueous suspension.

### Nitrosation of MSA leading to S-nitroso-MSA

Both free and encapsulated MSA were nitrosated by reacting with equimolar amounts of sodium nitrite (NaNO_2_)^48^. An aqueous solution of MSA or an aqueous suspension of MSA-containing chitosan NPs (in both cases, MSA concentration of 10 mM) reacted for 1 h with NaNO_2_ in an ice bath protected from light. Under acidic pH, NaNO_2_ yields nitrous acid (HNO_2_) which, in turn, nitrosates MSA, leading to the formation of S-nitroso-MSA^30,48^.

2 $${{\rm{NO}}}_{2({\rm{aq}})}^{-}+{{\rm{H}}}^{+}\to {{\rm{HNO}}}_{2({\rm{aq}})}$$

3 $${{\rm{MSA}}}_{({\rm{aq}})}+{{\rm{HNO}}}_{2({\rm{aq}})}\to {\rm{S}}-{\rm{nitroso}}-{{\rm{MSA}}}_{({\rm{aq}})}+{{\rm{H}}}_{2}{{\rm{O}}}_{({\rm{l}})}$$

Hence, free and encapsulated S-nitroso-MSA were obtained. NPs with non-nitrosated MSA were also used as a control, since chitosan may induce diverse biological responses in plants, including some related to stress tolerance^43,44^. All formulations were diluted in water to 2 mM of MSA or S-nitroso-MSA and immediately used for the assays.

### Kinetics of NO release

The kinetics of NO release in the dark and under irradiation from both free and encapsulated S-nitroso-MSA were obtained by monitoring the spectral changes at 336 nm (ε = 980 mM^−1^ cm^−1^), which are associated with S-N bond cleavage and free NO release from S-nitroso-MSA^31,48^. The formulations were kept in the dark or under irradiation with a white-light irradiation system (model 41723–54, Cole Parmer, IL, USA) fixed at intensity 7 (which corresponds to 8.97 µmol m^−2^ s^−1^). Preliminary studies showed an average temperature of 38.5 °C of solutions kept for 3 h under full sun (data not shown). The kinetics data were acquired for 55–120 min with 5 min intervals at 38.5 °C using a 0.5-cm optical path quartz cuvette. The initial concentration of free or encapsulated S-nitroso-MSA was 2 mM. The quantity of NO released over time was calculated according to Beer’s law.

4 $${[{\rm{NO}}]}_{{\rm{t}}}=\frac{{A}_{0}-{A}_{t}}{0.5\times 980}$$

Equation 4 relates NO concentration at time t ([NO]_t_) to the absorbance of S-nitroso-MSA in the beginning of reaction (A_0_) and at time t (A_t_). The initial rates of NO release were calculated by the slope of linear regression of kinetic data from 0 to 15 min (R^2^ higher than 0.98). The steady state concentration was calculated as the average NO concentration after the establishment of the plateau.

### Plant material and growth conditions

The experiments with plants were performed in the outdoor nursery of the Laboratório de Biodiversidade e Restauração de Ecossistemas, Universidade Estadual de Londrina, Londrina, PR, Brazil (23°19'28″S, 51°11′54″W). Neotropical tree species native to the Brazilian Atlantic Forest and belonging to distinct ecological groups were chosen for this study. *Heliocarpus popayanensis* Kunth (Malvaceae), a shade-intolerant pioneer species, is found in gaps and borders of primary forests and in secondary open forest formations. *Cariniana estrellensis* Raddi (Kuntze) is a shade-tolerant late successional species that develops in the understory of conserved forests but needs to reach the canopy to achieve reproductive maturity^49^. Seedlings of both species are commonly used in reforestation programmes in the neotropics.

The hardening routine conditions of the nursery were adopted in the study. Seeds were sown in sand, and the seedlings with the first completely expanded eophyll were transplanted to high-density polypropylene conical tubes of 50-cm^3^ volume containing a mixture of sieved plant organic matter and sand (9:1, v-v) supplemented with 3.5 g L^−1^ of a controlled release fertilizer (14% N, 14% P_2_O_5_ and 14% K_2_O). The seedlings were kept in the growth sector of the nursery under 40% of environmental photosynthetically active radiance until reaching approximately 15 cm of stem height. Then, the seedlings were transferred to the acclimation sector, where they were exposed to full sun (maximum PAR of approximately 1,800 µmol m^−2^ s^−1^) for six weeks. During the whole cultivation, the seedlings were watered four times daily for 30-min periods and exposed to natural precipitation (average daily precipitation of 11.2 mm) and temperature conditions (average, maximum and minimal temperatures of 23.9, 33.8 and 17.2 °C, respectively). The meteorological data were generously provided by Iapar – Instituto Agronômico do Paraná.

### Treatment of the plants with the formulations

The treatments performed on the seedlings are described in Table 3. The formulations (2 mL) were applied directly to the substrate just after the morning irrigation period (at 9 am). The treatments were carried out during three consecutive days immediately before the transfer to the acclimation sector followed by seven consecutive days at the beginning of full sun exposure. The treatments were repeated for seven days in the week before the end of the experiment.

**Table 3:** Treatments performed on *Heliocarpus popayanensis* and *Cariniana estrellensis* seedlings.

Treatment	Description
Control	Only water
S-nitroso-MSA NPs	Chitosan nanoparticles containing S-nitroso-MSA (2 mM)
S-nitroso-MSA	Non-encapsulated S-nitroso-MSA (2 mM)
MSA NPs	Chitosan nanoparticles containing MSA (2 mM)

### Growth measurements

Twelve seedlings were used for the evaluation of morphological parameters. After six weeks in the acclimation sector, the seedlings were removed from the tubes, and their roots were carefully washed in tap water. The diameter of the stem base (D) was measured using a digital calliper, while stem height (H) was determined with a ruler. Total leaf area was measured using a portable leaf area integrator LI-3000CAP (LiCor Inc., Lincoln, NE, EUA). For the leaf, stem and root dry mass determination, the organs were dried at 60 °C until reaching constant weight. Specific leaf area was calculated by the ratio between total leaf area and leaf dry mass. Shoot dry mass was obtained from the sum of root and stem dry masses, and the total dry mass of the seedlings (TDM) was obtained as the sum of root and shoot dry masses. Indexes related to seedling quality were also calculated, such as shoot-to-root dry mass ratio (S/R), height/diameter ratio (H/D) and Dickson’s quality index (DQI = TDM/(H/D + S/R))^50^.

### Physiological measurements

Ten seedlings were used for the evaluation of gas exchange parameters on the day before the end of the experiment. The net photosynthetic rate (*A*) of the youngest fully expanded leaf was measured between 8 and 10 am on a sunny day using a Portable Photosynthesis System LICOR 6400 XT (LiCor Inc., Lincoln, NE, EUA). The infrared gas analyser was connected to a 6-cm² measuring chamber with a flow rate of 400 mL min^−1^ and ambient CO_2_ concentration. The LED light source (680 nm) was set to 1,900 μmol m^−2^ s^−1^, enough to saturate the photosynthesis of both species^51^.

### Biochemical measurements

At the end of the experiment, the youngest fully expanded leaves of five seedlings were sampled for biochemical analyses. For all analyses, leaves were ground to a powder in liquid nitrogen before extraction.

For the determination of H_2_O_2_, leaves (0.1 g) were extracted with 1 mL of trichloroacetic acid (0.2% in methanol) and centrifuged at 15,644 xg for 15 min at 4 °C. H_2_O_2_ content was measured at 390 nm after the reaction with KI (1 M) in phosphate buffer (0.1 M, pH 7.5), following the methodology proposed by Alexieva *et al*.^52^.

For the analysis of the activity of antioxidant enzymes, leaves (0.1 g) were extracted with a medium composed of phosphate buffer (0.1 M, pH 7.5), EDTA (1 mM) and PVPP (2%). After centrifugation of 15,644 xg at 4 °C for 15 min, 100-μL aliquots of the extract were stored at −80 °C. Superoxide dismutase (SOD) activity was estimated by evaluating the extent of inhibition of nitroblue tetrazolium photoreduction by leaf extracts^53^. Ascorbate peroxidase (APX) activity was measured following ascorbate consumption at 290 nm in the presence of H_2_O_2_ ^54^. Peroxidase (POD) activity was evaluated following purpurogallin formation from pyrogallol at 420 nm in the presence of H_2_O_2_ ^55^. The protein content in leaf extracts was determined using the Comassie Blue reagent from Bio-Rad, following the manufacturer’s instructions.

Leaves (0.1 g) were extracted with methanol (80%) for the quantification of phenols and flavonoids. The total content of phenolic compounds was determined with Folin-Ciocalteau reagent^56^. The total flavonoid content was measured at 425 nm following the procedure described by Lee *et al*.^57^.

Leaf RSNO content was determined using the amperometer WPI TBR4100/1025 with a sensor ISO-NOP 2 mm (World Precision Instruments Inc., Sarasota, FL, USA), as described by Oliveira *et al*.^30^.

### Statistical analysis

The experimental design was completely randomized. Data were checked for normality by the Shapiro-Wilk test and for homoscedasticity by the Bartlett test. When necessary, data were transformed by the box-cox function. Data were submitted to analysis of variance (ANOVA), and when significant, the averages were compared by the Tukey test at the 5% probability level. All analyses were performed in R software, version 3.5.1.

## Data Availability

The authors declare that all data supporting the findings of this study are available within the paper.
